# Dynamics of Intrinsic Dendritic Calcium Signaling during Tonic Firing of Thalamic Reticular Neurons

**DOI:** 10.1371/journal.pone.0072275

**Published:** 2013-08-21

**Authors:** Patrick Chausson, Nathalie Leresche, Régis C. Lambert

**Affiliations:** 1 UMR 7102 CNRS, Paris, France; 2 UPMC, Université Paris 6, Paris, France; University of South Alabama, United States of America

## Abstract

The GABAergic neurons of the nucleus reticularis thalami that control the communication between thalamus and cortex are interconnected not only through axo-dendritic synapses but also through gap junctions and dendro-dendritic synapses. It is still unknown whether these dendritic communication processes may be triggered both by the tonic and the T-type Ca^2+^ channel-dependent high frequency burst firing of action potentials displayed by nucleus reticularis neurons during wakefulness and sleep, respectively. Indeed, while it is known that activation of T-type Ca^2+^ channels actively propagates throughout the dendritic tree, it is still unclear whether tonic action potential firing can also invade the dendritic arborization. Here, using two-photon microscopy, we demonstrated that dendritic Ca^2+^ responses following somatically evoked action potentials that mimic wake-related tonic firing are detected throughout the dendritic arborization. Calcium influx temporally summates to produce dendritic Ca^2+^ accumulations that are linearly related to the duration of the action potential trains. Increasing the firing frequency facilitates Ca^2+^ influx in the proximal but not in the distal dendritic compartments suggesting that the dendritic arborization acts as a low-pass filter in respect to the back-propagating action potentials. In the more distal compartment of the dendritic tree, T-type Ca^2+^ channels play a crucial role in the action potential triggered Ca^2+^ influx suggesting that this Ca^2+^ influx may be controlled by slight changes in the local dendritic membrane potential that determine the T-type channels’ availability. We conclude that by mediating Ca^2+^ dynamic in the whole dendritic arborization, both tonic and burst firing of the nucleus reticularis thalami neurons might control their dendro-dendritic and electrical communications.

## Introduction

Back-propagation of action potentials (APs) in the dendritic tree has been extensively studied in a number of neurons where it has been shown to determine the neuronal firing pattern, to contribute to dendritic integration and to support long-term and spike-timing dependent plasticity [1]. Moreover, by evoking widespread Ca^2+^ signals throughout the dendritic arborization, back-propagating APs are also likely candidate for dendritic neurotransmitter release at dendro-dendritic synapses [2] (see also review by [3]). The physiological consequences of dendro-dendritic synapse activation have been analyzed in details in the olfactory bulb [4] and, although far less investigated, dendro-dendritic synapses involving GABAergic neurons have also been described in the thalamus. In the lateral geniculate nucleus, interneurons express GABAergic vesicles in dendritic appendages and a majority of the interneuron synapses on the thalamocortical neurons are made by dendritic boutons [5], [6]. Combining whole-cell recordings and either two-photon Ca^2+^ imaging [7] or voltage-sensitive dye imaging [8], it has been shown that APs of interneurons back-propagate with high fidelity through the dendrites contributing to GABA release and feedforward inhibition of thalamocortical neurons.

In the Nucleus Reticularis Thalami (NRT), a GABAergic nucleus that controls the communication between the thalamus and the cortex and plays a crucial role in the generation of the synchronized activities within the thalamocortical loop during sleep, neurons are interconnected not only through axo-dendritic synapses but also through gap junctions and dendro-dendritic synapses [9], [10]. NRT neurons present two modes of discharge according to the state of vigilance. During wakefulness, NRT neurons discharge tonically but they switch to a high frequency bursting mode underlied by a low-threshold Ca^2+^ spike (LTS) during low vigilance and sleep [11]. Generation of this LTS is due to the recruitment of the T-type Ca^2+^ channels following their de-inactivation by hyperpolarization. It has been recently shown that activation of T-type Ca^2+^ channels underlying the high frequency burst firing actively propagates throughout the dendrites suggesting that the bursting mode of firing may support dendro-dendritic communication [12]. However, whether tonic AP firing can also invade the dendritic arborization remains unclear.

Here, using two-photon microscopy, we investigated the dynamics of intrinsic dendritic Ca^2+^ signaling across the NRT dendritic tree. We found that dendritic Ca^2+^ responses following somatically evoked APs can be detected in the dendritic arborization of the NRT neuron in thalamic slices at physiological temperature. In the more distal compartment of the dendritic tree, T-type Ca^2+^ channels play a crucial role in the action potential triggered Ca^2+^ influx suggesting that this Ca^2+^ influx may be controlled by slight changes in the local dendritic membrane potential that determines the T-type channel availability. Therefore, we conclude that both tonic and burst firing trigger intracellular Ca^2+^ increase throughout the NRT neuron arborization potentially linking neuronal firing to the dendritic integration and communication processes.

## Methods

### Ethical Approval

Ethical approval was obtained for all experimental protocols from the Departmental Direction of Veterinary Services, Paris. All procedures involving experimental animals were carried out in accordance with the EU Council Directive 86–609. Every effort was made to minimize animal suffering and the number of animals used. For removal of tissues, animals were deeply anesthetized with inhaled isoflurane and immediately sacrificed.

### Preparation of Brain Slices and Recordings

Brains were excised from 12–18 day old Wistar rats. A block of tissue containing the thalamus was removed, placed in a cold (<4°C) oxygenated (95%O_2_/5%CO_2_) solution of artificial cerebrospinal fluid (aCSF) (in mM): 125 NaCl, 2.5 KCl, 0.4 CaCl_2_, 1 MgCl_2_, 1.25 NaH_2_PO_4_, 26 NaHCO_3_, 25 glucose, and 1 kynurenic acid (pH 7.3; osmolarity 310 mOsm). The block of tissue was glued, ventral surface uppermost, to the stage of a vibroslice (Leica VT1200S), and 220–300 µm thick horizontal slices containing the ventrobasal nucleus and the NRT were prepared using the internal capsule and the medial lemniscus as landmarks. Slices were stored in an oxygenated incubation chamber containing aCSF of the above composition, but without kynurenic acid and with 2 mM CaCl_2_, for at least 1 h before being transferred to the recording chamber, where they were perfused (2.5 ml/min) continuously with an oxygenated recording solution of the same composition. Experiments were conducted at 32°C.

Using the patch-clamp technique (Axopatch 200B; Clampex 10, Molecular Devices), whole-cell recordings in current clamp mode were performed in NRT neurons visualized with an Olympus BX51WI (x60 lens). Recordings were filtered by a 4-pole Bessel filter set at a corner frequency of 1 kHz and digitalized at 10 kHz and latter analyzed using Matlab R2009b (The MathWorks, Inc.).

Electrodes were filled with the following solution (in mM): 140 methanesulfonic acid, 4 MgCl_2_, 10 HEPES, 4 Na-ATP, 15 phosphocreatine, 150 units/ml creatine phosphokinase; pH 7.3, osmolarity 280 mOsm. Alexa Fluor 594 and Oregon Green 488 Bapta-1 (Molecular Probes) were added with a final concentration of 15 and 100 µM, respectively (tip resistances: 1.8–2.4 MΩ; access resistance: 5–15 MΩ). At least 70% of the cell capacitance and series resistance were compensated. The liquid junction potential (+6 mV) was systematically corrected.

Experiments were performed in the presence of CNQX (10 µM) to block AMPA and kainate receptors. Action potentials and LTS were evoked by somatic current injections using either 100–700 pA, 5 ms square pulses (for APs) or 100–300 pA, 30 ms pulses (for LTS), respectively. Membrane potential was held at −50 to −60 mV for AP generation and −75 to −80 mV for LTS generation by constant current injection. Stimulus trials were delivered at 20–30 s intervals.

### Calcium Imaging

Images were obtained with a custom-built 2-photon laser scanning microscope. Two-photon excitation of the fluorescent dyes was performed by a femtosecond Ti:Saphir laser (Mai Tai HP, Spectra-Physics, Mountain View, CA) tuned to 800 nm. Image acquisition was controlled by MPScope software [13]. Before imaging, neurons were loaded with indicators for 30 min. The fluorescent signals from Alexa Fluor 594 and Oregon Green were acquired simultaneously across dendrites at selected regions of interest (ROI) (acquisition frequency = 40–60 frame/s) by two high gain photomultiplier tubes (PMT Hamamatsu H9305-03, Hamamatsu photonics, Japan). At the end of the recordings Z series of 160–190 images (512 pixels, 0.26 µm/pixel) were taken with 0.5 µm focal steps to construct a two-dimensional maximum intensity projection of each neuron.

### Data Analysis

Recordings were analyzed off-line using Matlab R2009b (The MathWorks, Inc.). Morphological distances were approximated by measuring along the dendrite from the beginning of the dendrite to the ROI of interest on the two-dimensional maximum intensity projection of each neuron. Fluorescence signals were measured by integration of the signal over a region of interest. The reported change in fluorescence (ΔG/R) was calculated as the change in fluorescence (Gpeak) from baseline (G0, average of the 400 ms period before stimulus) of the Ca^2+^-sensitive indicator (Oregon Green 480 Bapta-1) normalized to the average fluorescence of the Ca^2+^-insensitive indicator (RAvg, Alexa Fluor 594): ΔG/R = (G_Peak_−G_0_)/R_Avg_ [14]. The peak response was calculated as the combined average of 10–20 trials to maximize signal-to-noise ratio.

Quantitative data in the text and figures are given as mean ± s.e.m. Two-tailed t-tests were used to compare the values of ΔCa^2+^ evoked by LTSs. As action potentials ΔCa^2+^ values do not follow a normal distribution (Shapiro test), a non-parametric Wilcoxon-Mann Whitney test was used to assess significance. Differences were considered significant for p<0.05.

### Drugs

TTA-P2, 3,5-Dichloro-N-[1-(2,2-dimethyl-tetrahydro-pyran-4-ylmethyl)-4-fluoro-piperidinx-4-ylmethyl]-benzamide, was made up as 10 mM stock solution in dimethyl sulphoxide, kept at −20°C until use [15], [16]. TTA-P2 was provided by Merck and Co., Inc. TTX was obtained from Latoxan, 6-cyano-7-nitroquinoxaline-2,3-dione (CNQX) from Sigma.

## Results

Combining whole-cell patch-clamp recordings with two-photon laser-scanning microscopy, we investigated NRT neuron Ca^2+^ dynamics in response to somatically generated tonic APs and LTS-elicited high-frequency burst discharges. Burst discharges were triggered from a membrane potential of −75 to −80 mV by short current injections of minimal amplitude. Similarly, trains of tonic APs were evoked by injecting brief depolarizing current pulses at a frequency of either 10 or 40 Hz from a membrane potential set between −50 and −60 mV where T-type Ca^2+^ channels are almost fully inactivated (see methods). Individual neurons were loaded through the patch pipette with both the Ca^2+^-sensitive fluorophore and a Ca^2+^-insensitive indicator to visualize the dendritic tree.

**Figure 1:**
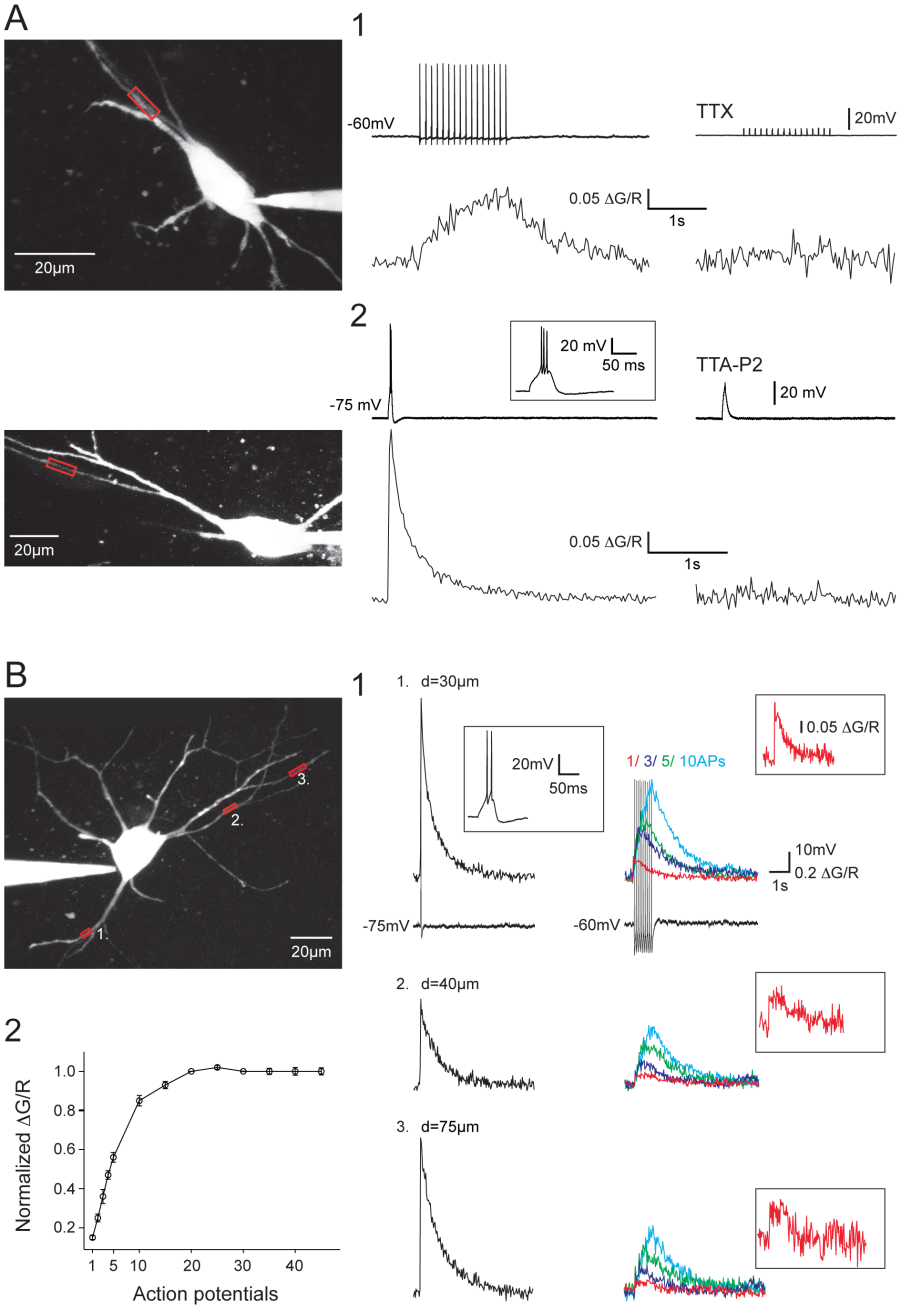
Dendritic Ca^2+^ responses evoked by tonic APs or LTS in NRT neurons. A1. Dendritic ΔCa^2+^ triggered by somatically evoked train of 15 APs at 10 Hz (top record). Application of 0.5 µM TTX fully blocked the APs and the ΔCa^2+^ (bottom trace). A2. Dendritic ΔCa^2+^ triggered by a somatically evoked LTS. An enlarged record of the LTS is presented in inset. Application of 3 µM TTA-P2 fully blocked the LTS and the ΔCa^2+^. B. ΔCa^2+^ recorded in three different dendrites of the same neuron. B1. Dendritic ΔCa^2+^ evoked by LTS (left column) and 1, 3, 5 and 10 APs at 10 Hz (right column) in the different dendrites. Examples of the somatically recorded voltage responses are shown underneath the ΔCa^2+^ recorded in location 1 (an enlarged record of the LTS response is illustrated in inset). ΔCa^2^ recorded in response to a single back-propagating AP is presented at higher magnification in inset. B2. Plot of amplitude of ΔCa^2+^ evoked by back-propagating APs (n = 10 neurons). The amplitude was normalized to the ΔCa^2+^ evoked at the time of the 20^th^ AP of the trains. Note the almost linear summation of the ΔCa^2+^ evoked by the first 5 back-propagating APs. In A and B the scanned dendritic regions are indicated by boxes on the maximal intensity Z projection of the neurons presented in the left panels.

**Figure 2:**
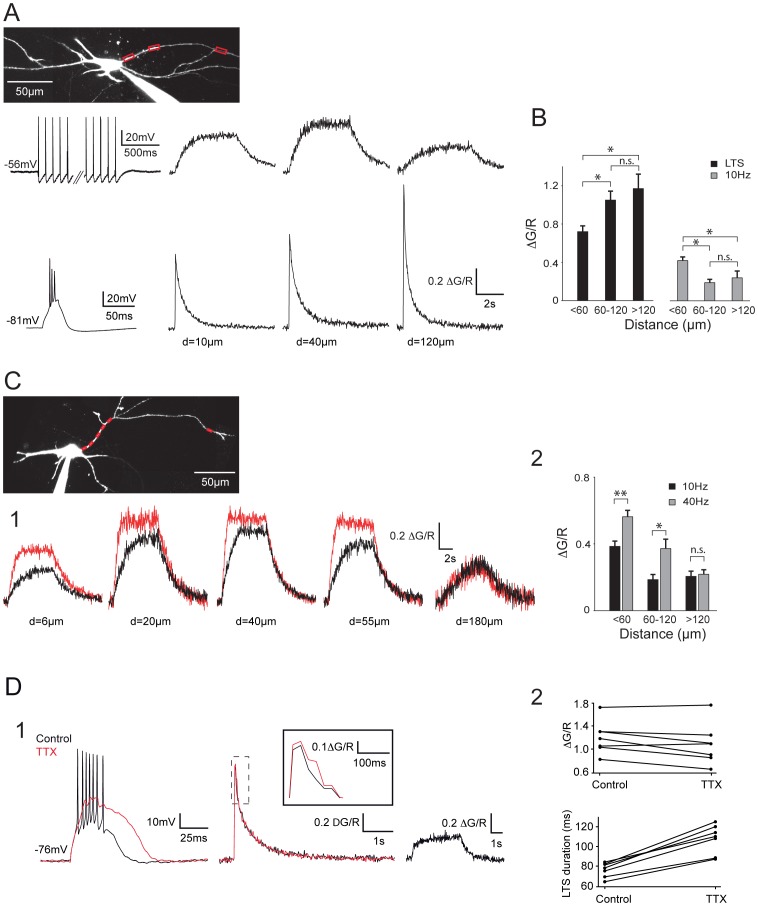
Back-propagating APs produce robust ΔCa^2+^ in distal dendrites of the NRT neurons. A. Top traces: ΔCa^2+^ recorded in response to a somatically evoked burst of 45 APs at 10 Hz (left trace). Amplitude of the ΔCa^2+^ first increased in the most proximal part of the dendrite (compare 10 and 40 µm) before decreasing in the more distal dendritic compartment. Note however that a noticeable ΔCa^2+^ is still present at the more distal location (120 µm). Bottom traces: ΔCa^2+^ recorded in response to a somatically evoked LTS (left trace). Note the increase in ΔCa^2+^ with dendritic distance. B. Summary of ΔCa^2+^ grouped by dendritic location. LTS: n = 19, 13 and 8; APs: n = 21, 15 and 6 for distances <60 µm, 60–120 µm and >120 µm, respectively. *: p<0.05, n.s.: p>0.05. C1. ΔCa^2+^ recorded in response to a somatically evoked burst of 45 APs at 10 (black traces) and 40 Hz (red traces). Increasing the firing frequency produced a clear enhancement in ΔCa^2+^ at proximal and intermediate locations but not at the more distal location (180 µm). C2. Summary of ΔCa^2+^ evoked in the same neurons by trains of 45 APs at 10 and 40 Hz grouped by dendritic location. <60 µm: n = 8; 60–120 µm: n = 7; >120 µm: n = 4. **: p<0.01, *: p<0.05, n.s.: p>0.05. D. Contribution of sodium APs to LTS-evoked dendritic ΔCa^2+^. D1. LTS (left traces) and associated dendritic ΔCa^2+^ (middle traces) measured at 110 µm from the soma in control condition and after TTX application. After blockade of the sodium APs, note the clear increase in amplitude and duration of the LTS and the lack of change in dendritic ΔCa^2+^. An enlargement of ΔCa^2+^ in both conditions is presented in inset. Right traces: at the same dendritic location a clear ΔCa^2+^ was evoked by a train of 45 APs at 10 Hz. Therefore, the lack of changes in LTS-evoked ΔCa^2+^ following TTX application did not result from the inability of APs to back-propagate in this distal dendrite. D2. Top plot shows the maximal fluorescence signal evoked in distal dendrites by LTS in control condition and after TTX application for each neuron. Note that TTX has no clear effect on calcium entry (mean ΔG/R: control 1.2±0.1, TTX: 1.08±0.13, p>0.05). Bottom plot presents the duration of LTSs in control condition and after TTX application (control: 76.1±2.8 ms, TTX: 107.4±5.6 ms, p<0.05). In A and C the scanned dendritic regions are indicated by boxes on the maximal intensity Z projection of the neurons.

Not only burst discharges but also single AP or trains of APs were able to transiently raise the intracellular Ca^2+^ concentration (ΔCa^2+^) in every NRT neuron dendritic branch (Figure 1B). Blocking sodium channels with TTX, or the T-type Ca^2+^ channels with the specific antagonist TTA-P2 [15] resulted in a full block of the ΔCa^2+^ evoked by single/tonic APs or burst firing, respectively (Figure 1A). As illustrated in Figure 1B, single AP triggered a small but clear increase in fluorescence in every dendrites that were monitored on the same neuron. The AP associated ΔCa^2+^ were present in both primary and secondary dendrites (Figure 1B1 and see also Figure 2C) and dendritic Ca^2+^ influxes temporally summated during 10 Hz trains of APs. Quantification of ΔCa^2+^ showed a linear relationship for the first 4–5 APs, reaching a plateau level around the 20^th^ AP of the train (Figure 1B2). This plateau was not due to the saturation of the Ca^2+^ sensitive dye since a LTS-elicited burst discharge always evoked a larger fluorescence increase at the same dendritic location.

To assess the extent of AP back-propagation in the dendritic tree of NRT neurons, the magnitude of the responses evoked by trains of APs at increasing distances from the soma was measured (Figure 2). Over the first tens of microns of the dendrite (<60 µm), the amplitude of the ΔCa^2+^ evoked by 10 Hz trains of APs either increased by 36±19% (n = 5, Figure 2), reaching a maximum between 20 and 40 µm, or remained stable (n = 8), when compared to the most proximal signal measured around 10 µm. In 6 neurons Ca^2+^ influxes could be monitored in both proximal and distal regions (120 to 200 µm) along the same dendrite. Although the AP evoked Ca^2+^ influx decreased in the distal dendrite, the Ca^2+^ response was still 54±10% of the maximal proximal response (Figure 2A). This mean value reflects a heterogeneous behavior as out of these 6 neurons, 4 of them presented a fairly small attenuation of ΔCa^2+^ with a distal signal corresponding to 67±9% of the Ca^2+^ influx observed in the proximal compartment while in the 2 remaining neurons, this percentage dropped to 35 and 23%, respectively. On average pooled data from all our results grouped by dendritic locations (n = 24 neurons, Figure 2B), clearly show that trains of APs can back-propagate up to the distal dendritic compartment in NRT neurons maintained at physiological temperature.

Increasing the frequency of firing to 40 Hz enhanced the ΔCa^2+^ plateau measured at proximal and intermediate dendritic locations (20–60 µm, 152±5% n = 8; 60–120 µm, 197±7% n = 7) (Figure 2C). As predicted from the temporal summation of Ca^2+^ influxes during AP trains, the increase in firing frequency also induced a faster Ca^2+^ accumulation (mean time constant: 742±120 ms and 278±40 ms for 10 and 40 Hz, respectively; n = 6). Surprisingly, accelerating tonic firing frequency from 10 to 40 Hz had no clear effect on the amplitude of ΔCa^2+^ in the more distal area (>120 µm; 104±1%; n = 4; Figure 2C) suggesting a strong low-pass filtering of the dendritic tree for back-propagating AP trains.

As previously reported by Crandall et al, [17], LTS mediated burst firing produced robust Ca^2+^ responses that increased along the dendrite (Figure 2A, 2B). In the proximal part of the dendrites (<60 µm) the ratio of ΔCa^2^ due to trains of 10 Hz action potentials versus LTS could be close to 1 or even larger in some neurons (1.21±0.12; n = 5), while in the remaining neurons it was around 0.5 (0.45±0.04; n = 15). However due to the inverse spatial relationship between the magnitudes of ΔCa^2^ evoked by an LTS and back-propagating APs, respectively, this ratio rapidly dropped to a value of 0.2 for dendritic distances greater than 60 µm (0.16±0.02; n = 18), increasing the difference between LTS- and AP-associated Ca^2+^ dynamics in the distal dendritic compartments.

Since trains of APs can back-propagate up to distal dendritic regions we then tested whether the high frequency bursts of APs contribute to the overall ΔCa^2+^ observed during LTS mediated firing. Although during bath application of 0.5 µM TTX, ΔCa^2+^ tend to decrease by 15.2±5.2 and 10.8±4.2% in the proximal (n = 5) and distal (n = 7) dendritic regions, respectively, these decreases were not significant (p>0.05) (Figure 2D). However the TTX effects are complex and blocking AP firing resulted in a larger and prolonged LTS (48.7±4.8% increase in total duration, n = 7; Figure 2D). Such change in LTS waveform that is likely due to the suppression of the strong AP after-hyperpolarization, may affect LTS associated ΔCa^2+^ and impair isolation of the specific Ca^2+^ entry due to back-propagating APs.

**Figure 3:**
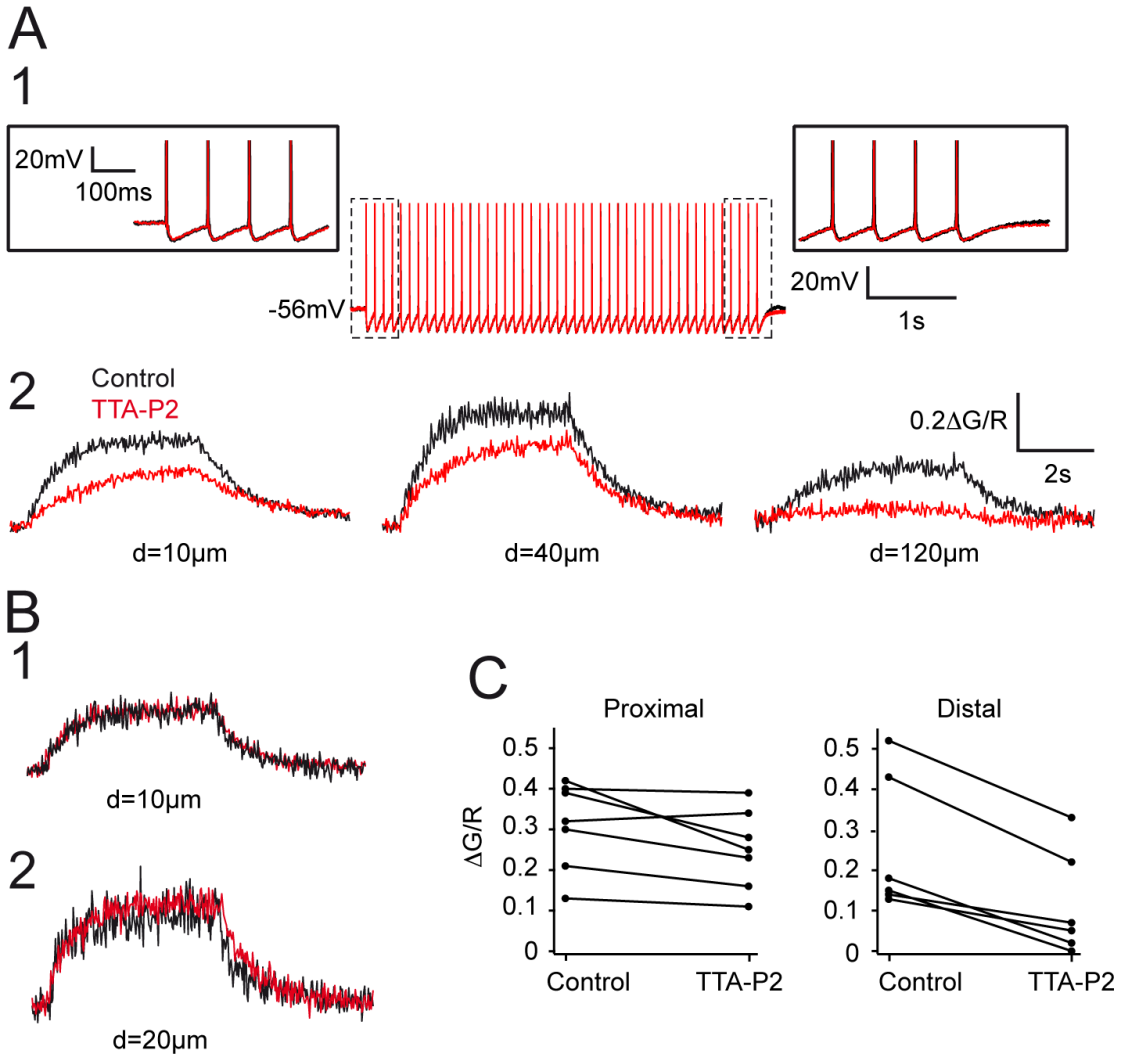
Contribution of T-type current to AP back-propagation. A. APs (A1) and associated dendritic ΔCa^2+^ (A2) recorded in control condition and after TTA-P2 application (3 µM). Same neuron as in figure 2A. Note that TTA-P2 did not modify the tonic discharge of APs. Enlargements of the first and last APs of the train are presented in insets. In contrast, the block of the T-type current decreased the amplitude of ΔCa^2+^ and almost fully abolished Ca^2+^ entry in the distal dendritic region. B1–2. Examples of two neurons in which TTA-P2 had no effect on the ΔCa^2+^ recorded in proximal dendrites. Same calibration as in A2. C. Plots of the maximal fluorescence signal evoked by trains of APs in control condition and after TTA-P2 application for each neuron. The left graph shows that TTA-P2 has little effect on calcium entry monitored in proximal dendrites (mean ΔG/R: control 0.31±0.04, TTA-P2∶0.25±0.04, p>0.05). Right graph: same measurements performed on distal dendrites showed a clear decrease upon TTA-P2 application (mean ΔG/R: control 0.25±0.7, TTA-P2 0.11±0.05, p<005).

As T-type Ca^2+^ channels are assumed to be the major source of Ca^2+^ entry in the dendrites of the NRT neurons [17], we then investigated whether activation of these channels may also support the Ca^2+^ signal associated with the back-propagation of APs in distal dendrites. To test this hypothesis, we compared the back-propagation of AP trains in control condition and in the presence of TTA-P2. TTA-P2 did not modify the tonic firing evoked by trains of short current pulses and had no discernible effect on either the AP waveform or its after-hyperpolarization (Figure 3A1). In the more proximal dendrites TTA-P2 had either little or no effect in 5 out of 7 neurons (Figure 3B, 3C) while in the 2 remaining neurons, the ΔCa^2+^ was decreased by 30 and 40% (Figure 3A2, 3C). When considering the whole neuronal population TTA-P2 induced a mean non-significant decrease of 19.1±5.7% of the Ca^2+^ entry in the proximal dendrites (n = 7). In contrast, although at our holding potential set between −50 and −60 mV the T-type Ca^2+^ channel population should be almost fully inactivated, the selective T-type current antagonist markedly decreased ΔCa^2+^ monitored at dendritic location more distal than 80 µm (63.8±9.3%, n = 6) (Figure 3A2, 3C).

**Figure 4:**
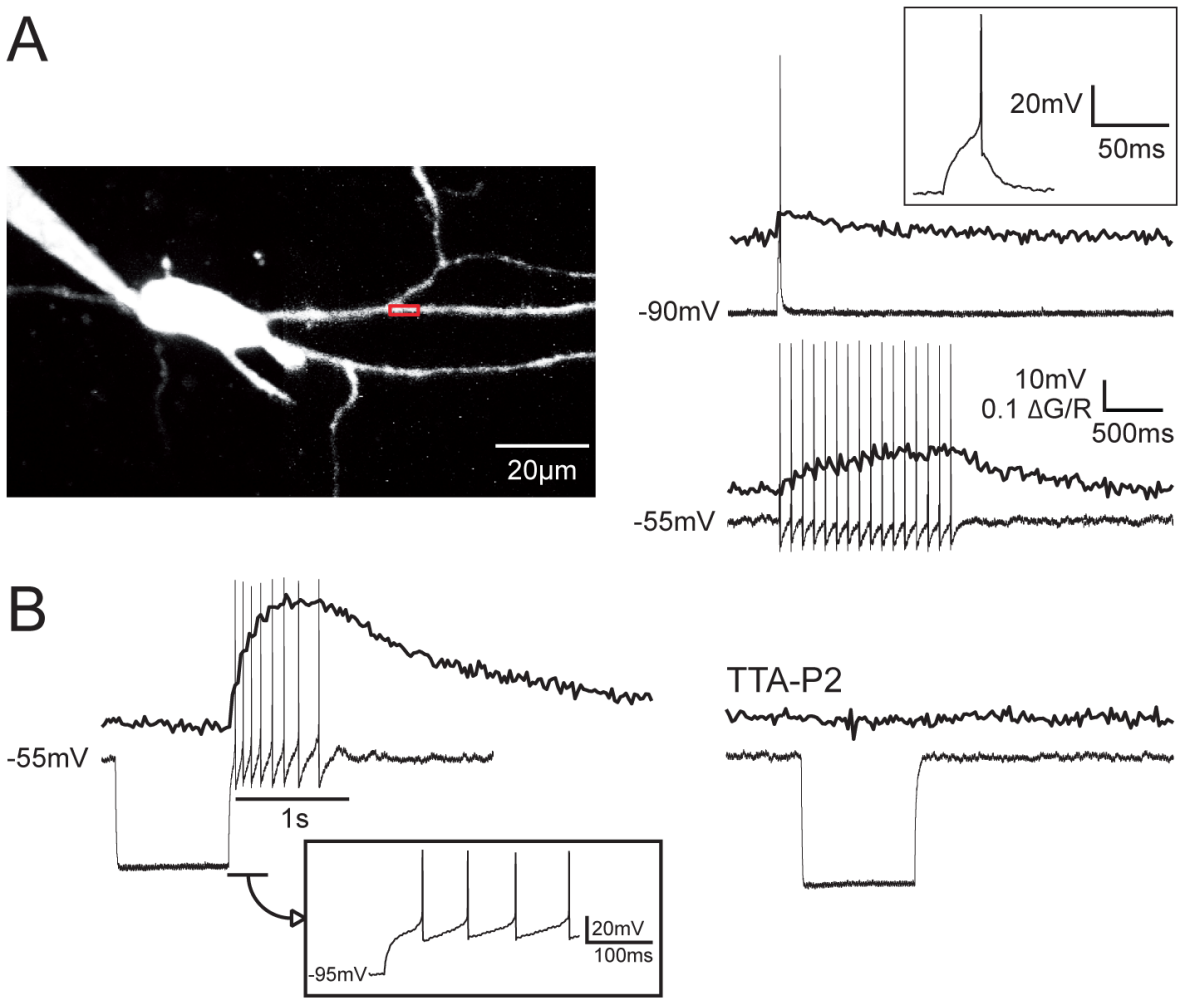
Dendritic ΔCa^2+^ in non-bursting NRT neuron. A. The scanned dendritic region is indicated by the box on the maximal intensity Z projection of the neuron (Left panel). Top traces show the dendritic ΔCa^2+^ and the voltage response to a depolarizing pulse from a holding potential of −90 mV. Note the lack of LTS and the presence of a single AP in the enlargement presented in inset. Bottom traces: Dendritic ΔCa^2+^ evoked by a train of 15 APs triggered from a −55 mV holding potential. B. A tonic discharge of 8 APs at 10 Hz was evoked as rebound activity by a 1 s hyperpolarizing pulse to −95 mV. Note the lack of LTS as clearly shown by the enlargement presented in inset. The dendritic ΔCa^2+^ associated to this tonic discharge is presented above. Both tonic discharge and the associated dendritic ΔCa^2+^ were abolished by application of 3 µM TTA-P2 (hyperpolarizing pulse from −55 to −100 mV). ΔG/R: same calibration as in A.

During the course of this study we patch-clamped three neurons in which no LTS could be evoked by injecting a depolarizing current step from hyperpolarized membrane potential (−80 to −90 mV) or after strong long-lasting hyperpolarizations that maximally recruit the T-type channels (Figure 4). However, a short train of tonic APs at a frequency around 10 Hz was observed at the break of the hyperpolarizing pulse associated to a clear ΔCa^2+^. Both the ΔCa^2+^ and the rebound tonic firing were blocked by TTA-P2, demonstrating that T-type Ca^2+^ currents are present in non-bursting NRT neurons where they participate to the generation of a tonic rebound activity. These neurons did not present a peculiar morphology when compared to other NRT neurons or a specific localization within the NRT.

## Discussion

The main finding of this study is that a tonic discharge of APs that mimics the firing of NRT neurons during wakefulness evokes a transient rise in intracellular Ca^2+^ that can be detected up to the distal regions of their dendrites (200 µm) and that T-type channels play a crucial role in this distal dendritic Ca^2+^ influx.

Using a similar approach, Crandall et al [17] previously reported that a somatically-evoked LTS produces a transient Ca^2+^ response along the NRT neuronal dendrites, that increases in magnitude at greater distances from the soma (up to 200 µm). However, in contrast to our data, single, or trains of, APs produced a minimal Ca^2+^ influx that was restricted to the most proximal dendrites (15 to 25 µm). A main difference between these two studies is the temperature at which experiments were performed, with Crandall and al. [17] being carried out at room temperature while we used a more physiological temperature (32–34°C). It is well known that temperature drastically modifies the biophysical properties of ionic channels, including the T-type Ca^2+^ channels [18], and therefore should condition both active and passive properties of the dendritic tree with major consequences on its ability to back-propagate APs and/or to generate Ca^2+^ influxes. In addition, different tonic firing patterns were used in the two studies. Crandall et al. [17] investigated the back-propagation of a short train of 4 APs at 100 Hz that mimics the burst of APs crowning an LTS rather than the tonic firing of NRT neurons. During waking, NRT neurons have a mean firing rate of 7–22 Hz in head-restrained cat [19] and of about 40 Hz in freely moving cats [20]. Moreover, in anesthetized cats, NRT neurons fire tonically at a frequency of 30 Hz in response to sensory stimulation [21]. To match the firing of NRT neurons during wakefulness, our protocols consisted of 15 to 45 APs at a frequency of either 10 or 40 Hz. Using these protocols, we showed that increasing the firing frequency facilitates Ca^2+^ influx in the proximal but not in the distal dendritic compartment suggesting that the dendritic arborization acts as a low-pass filter for back-propagating APs. This filtering ability should also dampen the propagation of short high-frequency trains of APs such as the ones used in Crandall et al., [17]. Accordingly we did not observe any consistent decrease in Ca^2+^ influx when blocking the high frequency burst of APs with TTX during a somatically evoked LTS. However, this result should be considered with caution since blocking APs firing induces marked changes in the LTS waveform that may mask a decrease in ΔCa^2+^ due to the loss of the high-frequency back-propagating APs.

In agreement with previous studies [22], [23], [24], we report that a small proportion of NRT neurons does not display high-frequency burst. Such inability to trigger LTS following somatically imposed membrane hyperpolarization is likely due to a reduced density of T-type channels in these cells, at least in their somatic and proximal dendritic compartments. However, transient rebound tonic activities, fully blocked by TTA-P2, were observed in these neurons demonstrating the presence of a minimal amount of T-type channels. This surprising rebound tonic activity exceeds the duration of T-type current mediated depolarization suggesting the activation of an additional transient depolarizing conductance, such as the Ca^2+^ activated non-selective cationic (CAN) current [25], [26]. Nevertheless, nearly all NRT neurons express a high density of T-type channels and our results indicate that back-propagation of APs depends in part upon activation of these channels since a clear decrease of ΔCa^2+^ was observed following TTA-P2 application. The contribution of T-type current to the Ca^2+^ influx associated to AP back-propagation was not constant along the entire dendritic tree since the TTA-P2 effect was more pronounced in distal than in proximal/intermediate dendritic compartment. This spatially gradual effect may reflect an attenuation of the amplitude of the AP-evoked depolarization along the dendrite that would become too small to activate high-voltage activated Ca^2+^ currents in the distal dendritic compartments. Alternatively, the expression of the high-voltage activated Ca^2+^ channels may be restricted to the more proximal dendrites and the T-type channels may constitute the only active Ca^2+^ channels expressed in distal dendritic branches. Accordingly, morphological data confirm the dense distribution of T-type channels on the dendritic membrane of NRT neurons [27], and experimental and computational data suggested a higher density of T-type currents in distal dendrites [28]. The contribution of T-type channels to the Ca^2+^ influx evoked by back-propagating APs is at first glance surprising since tonic activity was triggered from a holding potential set between −50 to −60 mV where T-type channels are thought to be almost fully inactivated [29], [30], [31]. However the membrane potential of the distal dendritic compartment may not be fully controlled by the somatic electrode [32]. As a consequence, a hyperpolarizing gradient of the membrane potential may exist between the soma and the distal dendrites with a similar increasing gradient of deinactivated T-type channels. Nevertheless, because in some NRT neurons T-type currents clearly participated to the Ca^2+^ influx in the first tens microns of the dendritic branch (see Figure 3A2), it is unlikely that the T-type current activation can be fully explained by a lack of control of the dendritic membrane potential by our somatic electrode. We have previously demonstrated that, due to the high-density of T-type channels expressed in thalamic neurons, the small fraction of deinactivated channels at depolarized potential is responsible for a physiologically meaningful current. Indeed, although the open probability of the channels is low at −60 mV, the number of available channels in both thalamocortical and NRT neurons is sufficient to generate a window current that contributes to the resting membrane potential of these neurons [15]. Moreover, in thalamocortical neurons maintained at depolarized potentials, we recently demonstrated that the drastic increase in open probability induced by sensory excitatory inputs induces a significant T-type current that boost the post-synaptic excitatory potential [33]. Therefore, back-propagating APs may similarly recruit the deinactivated channels population that is present at depolarized potentials in the NRT neurons to generate a clear Ca^2+^ influx. Nevertheless, the major role played by T-type current in dendritic Ca^2+^ dynamics during tonic firing suggests that this Ca^2+^ signal should be closely controlled by both excitatory and inhibitory synaptic inputs that influence local dendritic potential.

Conversely, dendritic Ca^2+^ influxes triggered by APs may control integration of the incoming inputs in NRT neurons. Indeed, among the classical roles attributed to back-propagating APs is their function in synaptic plasticity. Glutamatergic synapses impinge on NRT neurons with an organized pattern, as thalamocortical inputs mainly innerve the soma and proximal dendrites [34], [35], [36] while the corticothalamic synapses mostly contact the intermediate or distal dendritic compartments [37]. GABAergic synapses arising from intra-NRT connections impinge on the whole dendritic arborization [38], [39]. Therefore, by showing clear Ca^2+^ influxes not only in the proximal and intermediate dendritic regions but also in the more distal dendritic branches, our results suggest that back-propagating APs evoke local signals at corticothalamic, thalamocortical and intra-NRT GABAergic post-synaptic areas that may trigger short or long-term modification. In addition, as highlighted in the introduction, back-propagating APs could not only contribute to the integration of incoming synaptic inputs but may also participate to the activation of dendro-dendritic synapses [2], [3]. Finally, NRT neurons are strongly coupled by electrical synapses composed of connexin36 proteins [40]. It was recently shown that LTSs induce an LTD of the electrical coupling between NRT neurons [41]. Although delayed and of reduced amplitude, this LTD could also be induced by paired sodium spikes evoked at depolarized potentials, and it is highly likely that the increase in intracellular Ca^2+^ through back-propagating APs is required to trigger plasticity of the electrical synapses. Therefore, by mediating Ca^2+^ dynamic in the whole dendritic arborization, tonic firing of NRT neurons may control their dendro-dendritic and electrical communications as well as the integration of their synaptic inputs.
